# Atomistic Study of Polystyrene Supported by Amidinium-Based Ionic Liquid for CO_2_ Absorption

**DOI:** 10.3390/molecules31132360

**Published:** 2026-07-04

**Authors:** Irina Irgibaeva, Anuar Aldongarov, Lyazzat Abulyaissova, Abzal Taltenov, Damen Nurgaliyeva, Mirat Karibayev, Saparbek Tugelbay, Farkhad Tarikhov, Yerbolat Tashenov, Nikolay Barashkov

**Affiliations:** 1 Department of Chemistry, L.N. Gumilyov Eurasian National University, Astana 010000, Kazakhstan; abzal06@mail.ru (A.T.); tashenovyerbolat@gmail.com (Y.T.); 2 Department of Chemistry, Buketov National Research University, Karaganda 100028, Kazakhstan; abu.lyazzat@gmail.com; 3 Department of Natural Sciences, Astana International University, Astana 010000, Kazakhstan; damennurgalieva@gmail.com; 4 National Laboratory Astana, Nazarbayev University, Astana 010000, Kazakhstan; mirat.karibayev@nu.edu.kz (M.K.); saparbek.tugelbay@nu.edu.kz (S.T.); farkhad.tarikhov@nu.edu.kz (F.T.); 5 Micro-Tracers, Inc., 1370 Van Dyke Avenue, San Francisco, CA 94124, USA; nikolay@microtracers.com

**Keywords:** polystyrene, amidinium ionic liquid, CO_2_ absorption, density functional theory, non-covalent interaction analysis

## Abstract

The efficient capture of carbon dioxide (CO_2_) using polymer, supported ionic liquids (ILs) remains challenging due to limited understanding of atomic-scale interaction mechanisms. Here, a polystyrene (PS) oligomer supported by an amidinium chloride-based IL is proposed as a CO_2_-absorbing material. Density functional theory (DFT) calculations were employed to investigate the structural, electronic, and intermolecular interaction energy characteristics of the PS oligomer, amidinium chloride ILs, CO_2_, and their binary and ternary complexes. Molecular electrostatic potential maps (MEPs), reduced density gradient (RDG) plots with non-covalent interaction (NCI) snapshots, quantum theory of atoms in molecules critical point (CP) analysis, and electron localization function (ELF) analysis reveal pronounced hydrogen bonding and dispersion interactions between PS and IL that modulate the electronic environment of the IL anion, which is the primary CO_2_ binding site. Interaction energy calculations show that the ternary PS–IL–CO_2_ complex exhibits a significantly enhanced binding energy compared to the isolated IL–CO_2_ complex, providing quantitative evidence for the cooperative role of the PS support. The results indicate enhanced CO_2_ binding in the presence of PS supported by ILs, driven by cooperative electrostatic and dispersion interactions. These findings provide molecular-level insights into CO_2_ capture mechanisms in polymer–IL hybrid systems.

## 1. Introduction

The escalating concentration of atmospheric carbon dioxide (CO_2_) has intensified the search for efficient, reversible, and energy-sustainable capture technologies [1,2,3]. Among important materials, ionic liquids (ILs) have attracted considerable attention due to their negligible vapor pressure, high thermal stability, and tunable CO_2_ affinity [2,3,4]. However, the practical application of neat ILs is often hindered by high viscosity, limited gas diffusivity, and substantial material costs [3,4,5,6,7]. To address these limitations, polymer-supported ILs have been proposed as hybrid systems that combine the high CO_2_ selectivity of ILs with the mechanical robustness, processability, and surface area of polymeric matrices [8,9,10].

To contextualize the current state of research and highlight the diverse experimental and theoretical approaches employed, Table 1 summarizes representative recent studies on CO_2_ capture and fixation using hydroxy-containing amidine systems, along with their methodologies and key findings.

Table 1 highlights the evolution of hydroxy-containing amidine systems as efficient and versatile platforms for CO_2_ capture and fixation, spanning molecular, polymeric, and supported architectures [10,11,12,13,14,15]. Across the reported studies, a recurring theme is the central role of the amidine nitrogen as the primary nucleophilic site for CO_2_ activation, leading to the formation of zwitterionic, carbamate, bicarbonate, or alkyl-carbonate species depending on molecular structure and reaction environment. Notably, the presence of hydroxyl functionalities consistently enhances CO_2_ fixation by enabling cooperative hydrogen-bonding networks, which stabilize both the transition states and the resulting adducts.

While the experimental studies in Table 1 provide macroscopic evidence for CO_2_ capture and fixation by hydroxy-containing amidines, they do not fully elucidate the atomistic role of a supporting polymer matrix. Moreover, our previous atomistic investigation focused on hydroxy-containing amidinium-based ILs as standalone CO_2_ absorbers, revealing strong hydrogen-bonding networks and favorable charge distributions that enable spontaneous CO_2_ fixation [16,17]. While that study established the fundamental molecular mechanisms of amidinium–CO_2_ interactions, it did not account for the role of a supporting polymer matrix, which is essential for real-world applications requiring mechanical integrity and recyclability [18,19]. Polystyrene (PS) offers a particularly attractive support due to its low cost, hydrophobicity [17,18,19,20], and ability to engage in dispersion interactions with IL cations and anions, potentially modulating the electronic environment of the IL anion, the primary CO_2_ binding site.

To complement these observations, the present computational work investigates a polystyrene (PS) oligomer supported by a hydroxy-containing amidinium chloride IL. Our DFT-based predictions reveal that, unlike the isolated IL systems studied experimentally, the PS support actively participates in the capture mechanism. These cooperative effects result in a more stabilized ternary PS/IL/CO_2_ complex compared to the isolated IL, suggesting that polymer-supported systems can offer improved capture performance beyond the simple sum of their components. This molecular-level insight provides a rationale for the enhanced absorption capacities observed in hybrid polymer–IL materials and offers design principles for future experimental development.

In this work, we extend our previous DFT-based analysis to investigate a ternary system comprising a polystyrene oligomer supported by a hydroxy-containing amidinium chloride IL for CO_2_ absorption. We systematically examine the structural, electronic, and intermolecular interaction energy characteristics of the PS oligomer, the IL, CO_2_, and their binary and ternary complexes. Using molecular electrostatic potential (MEP) maps, reduced density gradient (RDG) analysis with non-covalent interaction (NCI) snapshots, quantum theory of atoms in molecules (QTAIM) critical point (CP) analysis, electron localization function (ELF) analysis, and interaction energy, we elucidate the cooperative hydrogen bonding and dispersion interactions between PS and the IL. Our results demonstrate that PS support enhances CO_2_ binding through cooperative electrostatic and dispersion interactions, offering molecular-level insights for designing polymer–IL hybrid capture systems.

## 2. Results and Discussion

### 2.1. Optimized Structures

**Figure 1:**
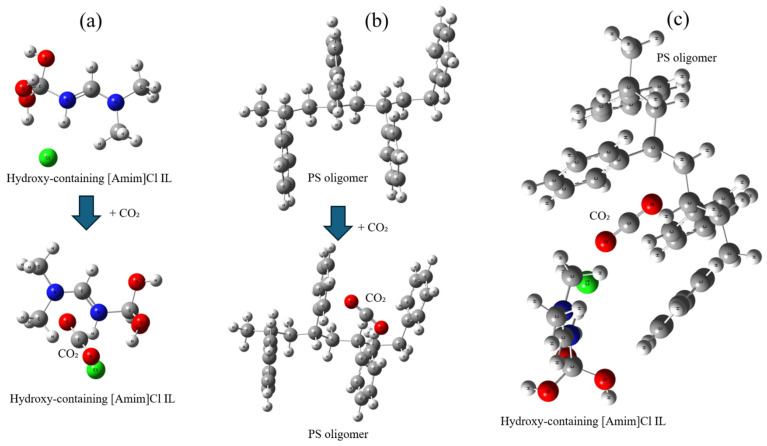
Optimized structure of (**a**) hydroxy-containing [Amim]Cl IL in the absence and presence of CO_2_, (**b**) PS oligomer in the absence and presence of CO_2_, and (**c**) PS oligomer supported by hydroxy-containing [Amim]Cl IL in the presence of CO_2_. Color scheme: dark gray—carbon; white—hydrogen; red—oxygen; blue—nitrogen; green—chloride.

Figure 1 illustrates the stepwise optimization of the ternary CO_2_ absorption system. Figure 1a shows the isolated hydroxy-containing [Amim]Cl ion pair. The chloride anion (Cl^−^) (green) is tightly bound to the amidinium cation via N-H⋯Cl hydrogen bonds, representing the “closed” resting state. Upon introduction of CO_2_, a dramatic structural rearrangement occurs. The Cl^−^ approaches the electrophilic carbon center of CO_2_, accompanied by a reorientation of the cation to stabilize the resulting bent CO_2_ geometry through additional N–H⋯O hydrogen bonds.

Figure 1b displays the pure PS oligomer in a coiled, compact conformation driven by C-H⋯π interactions between aromatic side chains. When CO_2_ is added, only weak physical adsorption is observed, CO_2_ remains isolated above the aromatic rings without bond formation, confirming PS acts as an inert, hydrophobic support rather than an active capture site.

Figure 1c reveals the critical ternary complex (PS/IL/CO_2_). Here, the IL binds non-covalently to the PS backbone through multiple Cl^−^⋯π and C-H⋯π contacts with the aromatic rings (Supplementary Material, Figure S1). This anchoring does not block but rather pre-organizes the IL. Compared to the isolated IL in Figure 1a, the PS-supported IL exhibits a more open geometry around Cl^−^, suggesting the polymer matrix modulates the electronic environment of the anion. Consequently, CO_2_ binding appears enhanced, with the bent CO_2_ geometry better stabilized by cooperative dispersion interactions from the nearby PS phenyl rings. These structural data indicate that PS actively tunes IL reactivity rather than serving merely as a passive carrier.

The structural parameters reveal distinct differences between the isolated IL–CO_2_ complex and the PS-supported system (Table 2). In our previous experimental and computational studies [16,17], we demonstrated that hydroxyl-containing amidines undergo CO_2_ fixation through zwitterionic alkyl-carbonate formation stabilized by hydrogen bonds (N-H⋯O hydrogen bonds of 2.18–2.25 Å).

In the present work, the IL–CO_2_ complex exhibits similar parameters (Cl⋯C = 3.22 Å, N-H⋯O = 2.22 Å), consistent with our earlier findings. Critically, the ternary PS–IL–CO_2_ complex shows a slightly shortened Cl⋯C distance (3.18 Å) and the emergence of Cl^−^⋯π (3.68 Å) and C-H⋯π (3.55 Å) interactions between the PS matrix and the IL. These cooperative non-covalent interactions, absent in the isolated IL system, demonstrate that PS actively modulates the IL’s electronic environment and enhances CO_2_ binding. Herein, the PS support provides an additional stabilization mechanism through dispersive interactions, leading to enhanced CO_2_ capture performance.

### 2.2. Molecular Electrostatic Potential Maps

**Figure 2:**
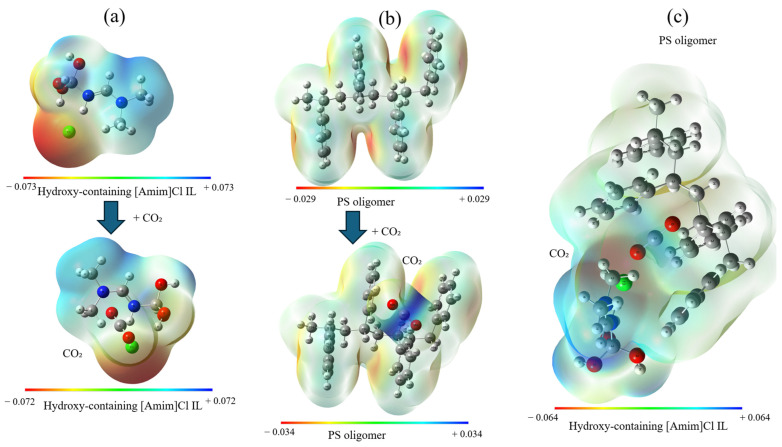
MEPs of (**a**) hydroxy-containing [Amim]Cl IL in the absence and presence of CO_2_, (**b**) PS oligomer in the absence and presence of CO_2_, and (**c**) PS oligomer supported by hydroxy-containing [Amim]Cl IL in the presence of CO_2_. Color scheme: dark gray—carbon; white—hydrogen; red—oxygen; blue—nitrogen; green—chloride. The isosurface values of the MEPs are illustrated in the figure using a color bar.

Figure 2 presents molecular electrostatic potential (MEP) maps to elucidate charge distribution changes upon CO_2_ binding and polymer support. Figure 2a shows the isolated [Amim]Cl IL. The amidinium cation exhibits intense blue (positive) regions localized on the N-H protons, indicating strong hydrogen-bond donor capacity. The chloride anion (green) displays a deep-red (negative) isosurface, confirming its high nucleophilicity as the primary CO_2_ binding site. Upon CO_2_ addition, the positive region on the cation becomes more diffuse, while the negative electrostatic potential lobe on the Cl^−^⋯CO_2_ interacting region redistributes, reflecting significant charge transfer from Cl^−^ to CO_2_.

Figure 2b depicts the PS oligomer. Its MEP is dominated by neutral to weakly negative (green–yellow) regions over the aromatic rings due to π-electron density, with slightly positive (light blue) areas on the aliphatic backbone hydrogens. CO_2_ alone shows characteristic negative (red) poles on the electronegative oxygens and a positive (blue) region on the electron-deficient carbon. When physisorbed onto PS, minimal MEP perturbation occurs, confirming purely dispersive interactions.

Figure 2c reveals the ternary PS/IL/CO_2_ complex (Supplementary Material, Figure S2). Critically, the negative potential on the Cl^−^-CO_2_ moiety is more pronounced and spatially extended compared to Figure 2a. Additionally, the aromatic rings of PS develop induced positive polarization (darker blue) facing the IL anion. This cooperative electrostatic environment suggests the polymer support enhances the anion’s nucleophilic character and stabilizes the bound CO_2_ through complementary charge–charge interactions, explaining the improved CO_2_ absorption observed experimentally.

### 2.3. Reduced Density Gradient and Non-Covalent Interactions

**Figure 3:**
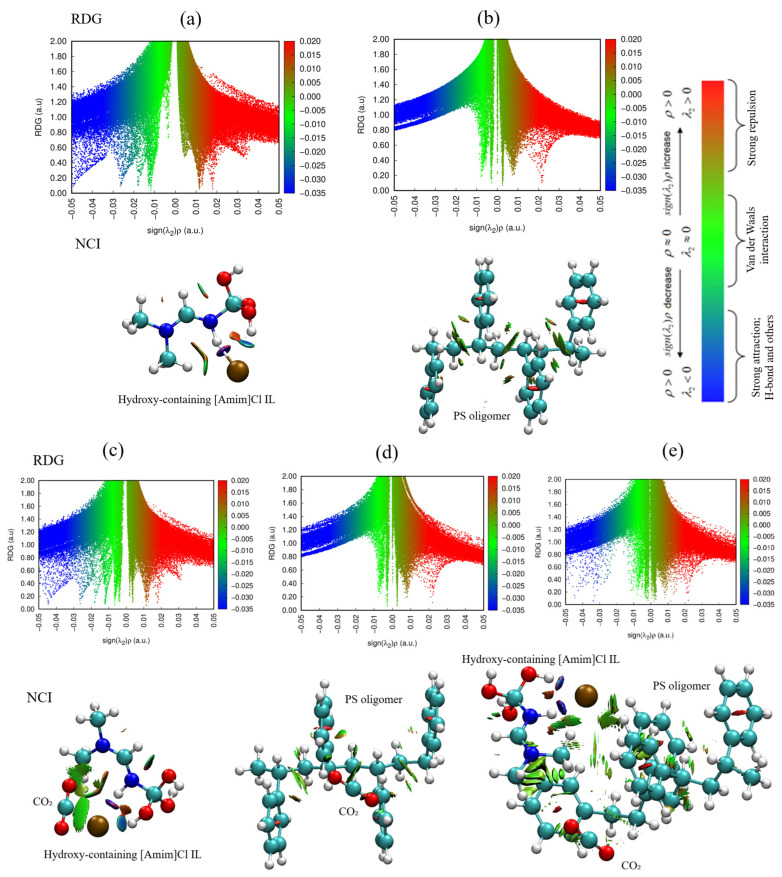
RDGs and NCIs of (**a**) hydroxy-containing [Amim]Cl IL, (**b**) PS oligomer, (**c**) hydroxy-containing [Amim]Cl IL in the presence of CO_2_, (**d**) PS oligomer in the presence of CO_2_, and (**e**) PS oligomer supported by hydroxy-containing [Amim]Cl IL in the presence of CO_2_. Color scheme: cyan—carbon; white—hydrogen; red—oxygen; blue—nitrogen; dark brown—chloride. The isosurface values of the NCI and RDG are illustrated in the figure using a color bar. Namely, color scheme of NCI (isosurface) and RDG: blue (strong attraction), green (van der Waals interaction), and red (strong repulsion). Various colored lines are bonds of atoms.

Figure 3 employs RDG versus sign(λ_2_)ρ scatter plots coupled with NCI isosurfaces to visualize and classify non-covalent interactions governing CO_2_ absorption. Figure 3a (isolated [Amim]Cl IL) reveals intense green disk-like isosurfaces between the chloride anion and amidinium N-H protons, characteristic of strong hydrogen bonding (sign(λ_2_)ρ ≈ −0.025 to −0.015 a.u.). Weaker green regions appear between the hydroxyl group and adjacent hydrogens, indicating secondary H-bonds.

Figure 3b (PS oligomer alone) shows extensive green–blue isosurfaces between aromatic rings and along the aliphatic backbone, corresponding to C-H⋯π and dispersion interactions typical of polymer chain collapse. No red (steric repulsion) or strong blue (attractive) regions are present, confirming PS’s non-polar, dispersive nature.

Figure 3c ([Amim]Cl IL + CO_2_) exhibits a dramatic change. The Cl^−^⋯H–N hydrogen bonds are partially disrupted, and a new, broad green isosurface appears between Cl^−^ and the CO_2_ carbon (sign(λ_2_)ρ ≈ −0.010 to −0.005). This feature is indicative of a strong, attractive non-covalent interaction, characteristic of a Cl^−^⋯CO_2_ close-contact configuration. Blue–green surfaces also appear between CO_2_ oxygens and amidinium N-H groups, signaling cooperative hydrogen-bond stabilization of the bent CO_2_ geometry.

Figure 3d (PS + CO_2_) displays only weak green isosurfaces at van der Waals contact distances between CO_2_ and aromatic rings, with no blue regions. This confirms purely physical, dispersive CO_2_ physisorption on PS.

Figure 3e (ternary PS/IL/CO_2_ complex) (Supplementary Material, Figure S3) reveals the most complex NCI network. Multiple green isosurfaces connect PS aromatic rings to both the IL cation and Cl^−^ anion, establishing a stabilizing “anchor” via Cl^−^⋯π and C-H⋯π interactions. Crucially, the Cl^−^-CO_2_ interaction region appears larger and more intense (shifted toward more negative sign(λ_2_)ρ) compared to Figure 3c, while additional blue–green surfaces appear between PS backbone hydrogens and CO_2_ oxygens. These observations provide direct visual evidence that the PS matrix cooperatively enhances IL-mediated CO_2_ binding through synergistic dispersion and electrostatic contributions.

### 2.4. Critical Points

**Figure 4:**
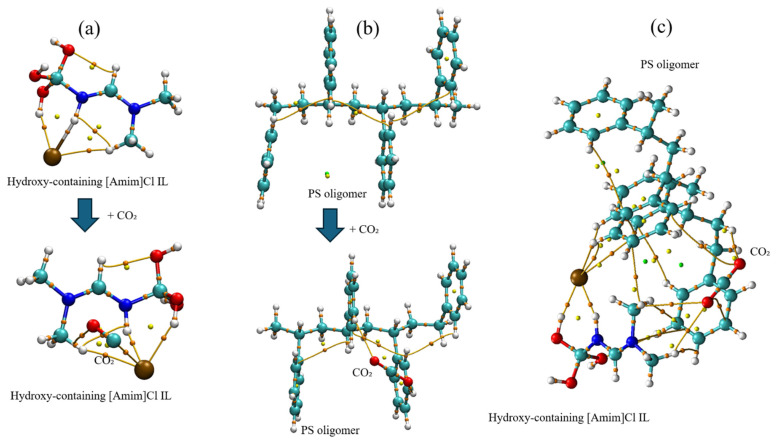
Critical points of (**a**) hydroxy-containing [Amim]Cl IL in the absence and presence of CO_2_, (**b**) PS oligomer in the absence and presence of CO_2_, and (**c**) PS oligomer supported by hydroxy-containing [Amim]Cl IL in the presence of CO_2_. Color scheme: cyan—carbon; white—hydrogen; red—oxygen; blue—nitrogen; dark brown—chloride. The yellow paths and small yellow and green dots in the figure indicate critical points.

Figure 4 presents bond critical points (BCPs, small yellow dots) and bond paths (yellow lines) from QTAIM analysis, enabling quantification of bonding and non-covalent interactions. Figure 4a (isolated [Amim]Cl IL) displays clear BCPs between the chloride anion and each amidinium N-H hydrogen, with bond paths tracing linear to slightly bent geometries. Additional BCPs appear between the hydroxyl O–H and adjacent nitrogen atoms, confirming an extended hydrogen-bonding network. The electron density (*ρ*(*r*)) at these Cl⋯H-N BCPs is relatively high (0.015–0.025 a.u.), characteristic of moderate-strength hydrogen bonds.

Upon CO_2_ addition (Figure 4a), new bond critical points (BCPs) emerge between Cl^−^ and the CO_2_ carbon atom, with bond paths tracing the interaction topology. This is consistent with a strong Cl^−^⋯CO_2_ interaction approaching the characteristics of a dative or charge-transfer stabilized contact.

Simultaneously, the original Cl⋯H-N BCPs weaken (lower *ρ*(*r*) values) or disappear, replaced by new BCPs between CO_2_ oxygens and amidinium N-H groups. This topological reorganization confirms CO_2_ insertion into the Cl^−^⋯cation hydrogen-bond network.

Figure 4b (PS oligomer) shows numerous BCPs between aromatic ring carbons and adjacent backbone hydrogens, representing C-H⋯π interactions with *ρ*(*r*) values around 0.005–0.010 a.u., typical for dispersion-dominated contacts. With CO_2_ added, weak BCPs appear between CO_2_ oxygens and phenyl ring hydrogens, but *ρ*(*r*) remains below 0.005 a.u., confirming physisorption.

Figure 4c (ternary PS/IL/CO_2_ complex) (Supplementary Material, Figure S4) reveals the most extensive CP network. New BCPs connect PS aromatic rings to the chloride anion (Cl^−^⋯π) and to the amidinium cation (C-H⋯π). Crucially, the Cl^−^-CO_2_ BCP exhibits higher *ρ*(*r*) (≈0.030 a.u.) than in the isolated IL, while additional BCPs appear between PS backbone hydrogens and CO_2_ oxygens. This topological enrichment provides unambiguous evidence that the PS support electronically enhances the primary CO_2_ binding site while contributing secondary stabilization through dispersive interactions.

### 2.5. Electron Localization Function

**Figure 5:**
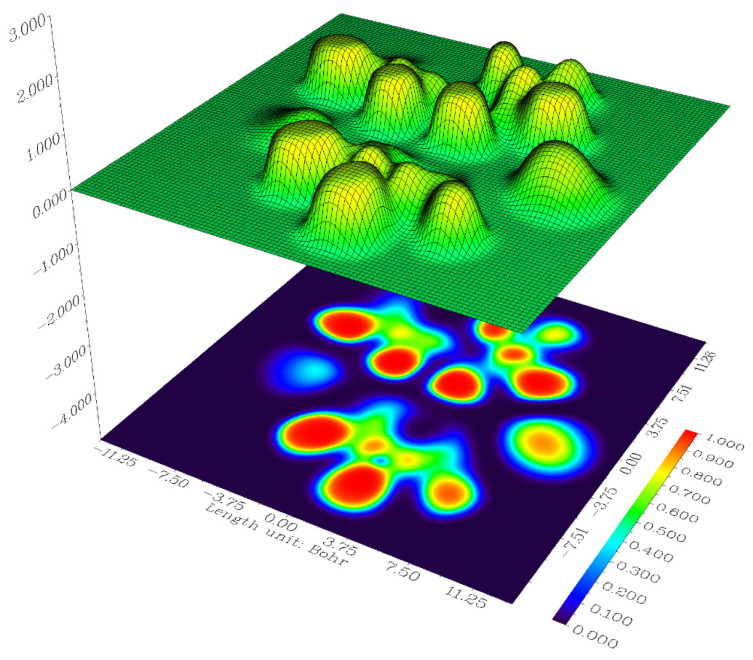
ELF of PS oligomer supported by hydroxy-containing [Amim]Cl IL in the presence of CO_2_.

Figure 5 presents the electron localization function (ELF) map of the ternary PS/IL/CO_2_ complex.

High ELF values (red–yellow regions) around the chloride anion and the CO_2_ carbon indicate significant electron localization, confirming covalent character in the forming Cl-CO_2_ bond. The amidinium N–H groups show localized basins directed toward CO_2_ oxygens, consistent with hydrogen-bond stabilization. Notably, the PS aromatic rings exhibit polarized electron density (moderate ELF) facing the IL, suggesting Cl^−^⋯π and C-H⋯π interactions that modulate the anion’s electronic environment. No electron delocalization occurs between PS and CO_2_ directly, confirming PS acts cooperatively rather than participating directly in CO_2_ binding.

### 2.6. Interaction Energies

To complement the structural and electronic analyses, we computed the interaction energies (*ΔE*) for the binary and ternary complexes at the B3LYP/6-311+G(d,p) level of theory (Table 3). As shown in Table 3, the IL–CO_2_ complex exhibits a *ΔE* of −13.72 kcal/mol, indicating favorable binding primarily through hydrogen bonding and electrostatic interactions. The PS–CO_2_ complex shows a significantly weaker *ΔE* of −1.75 kcal/mol, consistent with physisorption via dispersive interactions.

Remarkably, the ternary PS–IL–CO_2_ complex displays a *ΔE* of −35.00 kcal/mol, substantially more negative than the sum of the binary interactions. This cooperative enhancement provides quantitative evidence that the PS support actively stabilizes the CO_2_-bound state through synergistic Cl^−^⋯π and C-H⋯π interactions. We acknowledge that basis set superposition error (BSSE) corrections have not been applied; future work will incorporate these corrections for more accurate absolute binding energies. Compared to our previous studies on isolated hydroxyamidine–CO_2_ systems, which focused solely on neat ILs without polymer support [16,17], the present ternary complex exhibits a significantly enhanced interaction energy, demonstrating that the PS matrix plays an active role in CO_2_ binding.

In addition, the present study focuses on the hydroxy-containing [Amim]Cl ionic liquid; the observed cooperative interactions between the PS support and the ionic liquid are expected to depend strongly on the nature of the anion. Since chloride possesses relatively high basicity and nucleophilicity, it readily forms hydrogen-bonding networks and serves as the primary CO_2_ binding site, leading to enhanced stabilization of the ternary PS/IL/CO_2_ complex. Replacing chloride with less basic and weakly coordinating anions, such as tetrafluoroborate (BF_4_^−^) or hexafluorophosphate (PF_6_^−^), would likely reduce the strength of hydrogen bonding and nucleophilic interactions with CO_2_, thereby diminishing the cooperative enhancement provided by the PS support. In contrast, more basic anions such as acetate are expected to exhibit stronger interactions with CO_2_ and may further amplify the cooperative effects observed in this work. Systematic computational investigations of different anions will be the subject of future studies to establish broader structure–property relationships for polymer-supported ionic liquid CO_2_ capture systems.

## 3. Materials and Methods

### 3.1. Theoretical Model and Designed System

The molecular systems investigated in this study are illustrated in Figure 1. These systems were selected to capture both intrinsic molecular features and intermolecular interactions relevant to CO_2_ fixation. The presence of hydroxyl and amidine functional groups of IL enables simultaneous hydrogen bonding interactions with CO_2_, in the presence of PS oligomer.

In this study, a PS tetramer (four repeating units of styrene monomer) was employed to represent the polymer support. This PS oligomer length was selected based on established computational precedents for polymer–IL hybrid systems, where oligomers of 3–6 repeat units adequately capture local structural features and non-covalent interaction motifs relevant to polymer–small molecule binding [21,22]. The tetramer provides sufficient backbone flexibility and multiple aromatic side chains to enable the key Cl^−^⋯π and C-H⋯π interactions with the ionic liquid. It also captures the essential electronic environment of the PS repeat unit, including the electron-rich aromatic rings responsible for dispersive interactions. We acknowledge that this model does not fully represent macroscopic bulk properties such as chain entanglement and surface area. However, the cooperative non-covalent interactions identified here are local phenomena well-captured by the tetramer. Longer chains may introduce additional conformational effects but are not expected to fundamentally alter the binding motifs identified. Future studies employing larger oligomers and molecular dynamics simulations will be necessary to fully capture bulk effects.

### 3.2. DFT Calculations

Quantum-chemical calculations were carried out within the framework of density functional theory (DFT) to elucidate the molecular-level mechanisms governing CO_2_ fixation by PS supported by hydroxy-containing amidine ILs. All electronic structure calculations were performed using the Gaussian 16 software package [23], while molecular structures and visualization of optimized geometries were handled using GaussView 6.0 [24]. Geometry optimizations were conducted employing the three-parameter hybrid exchange–correlation functional Becke–Lee–Yang–Parr (B3LYP) [25] in conjunction with the 6-311+G(d,p) basis set [26], which provides a balanced description of valence and diffuse functions necessary for accurately capturing intermolecular interactions and weak non-covalent forces.

While the B3LYP functional with the 6-311+G(d,p) basis set does not explicitly include empirical dispersion corrections, it remains a well-established and extensively validated choice for studying ionic liquid systems where hydrogen bonding and electrostatic interactions dominate [9,16,17]. The inclusion of diffuse functions in the basis set is critical for accurately describing the anion’s electronic environment and the charge-transfer character of the Cl^−^⋯CO_2_ interaction. Importantly, our primary conclusions are derived from comparative analyses of electron density topologies (MEP, RDG/NCI, QTAIM, and ELF) rather than from absolute interaction energies. These wavefunction-based analyses are robust to the specific choice of functional, as they rely on electron density features that are well reproduced at the B3LYP/6-311+G(d,p) level for hydrogen-bonded and charge-transfer systems [27,28]. While we acknowledge that dispersion-corrected functionals would provide more accurate absolute binding energies, our comparative trends are expected to remain qualitatively robust. Future work will employ dispersion-corrected methods for quantitative validation.

Full geometry optimizations were performed without symmetry constraints using the opt freq keyword combination with geom=connectivity to preserve the bonding topology of the interacting species. Tight self-consistent field (SCF) convergence criteria of 10^−8^ a.u. were applied to ensure reliable electronic energy convergence. Frequency calculations were carried out at the same level of theory to confirm that the optimized structures correspond to true minima on the potential energy surface, characterized by the absence of imaginary frequencies.

The molecular systems investigated in this study comprised (i) the hydroxy-containing [Amim]Cl ionic liquid in the absence and presence of CO_2_, (ii) the PS oligomer in the absence and presence of CO_2_, and (iii) the ternary PS oligomer supported by the hydroxy-containing [Amim]Cl ionic liquid in the presence of CO_2_. All geometry optimizations and subsequent electronic structure analyses were performed in the gas phase (vacuum) to elucidate the intrinsic molecular interactions governing CO_2_ adsorption without introducing additional environmental effects. We acknowledge that practical CO_2_ capture occurs in condensed-phase polymer/ionic liquid systems, where dielectric screening and many-body interactions may influence the absolute interaction energies and structural characteristics. Nevertheless, the gas-phase calculations provide valuable mechanistic insight into the relative trends in CO_2_ binding and the role of the PS support in modulating the electronic environment of the ionic liquid. Future work will employ implicit solvent models and molecular dynamics simulations under realistic condensed-phase conditions to further validate these findings.

Key quantum-chemical descriptors extracted from the optimized structures include molecular electrostatic potential (MEP) maps, and other analyses.

The interaction energies were calculated as:

*ΔE* = *E* (complex) − [*E* (CO_2_) + *E* (IL) + *E* (PS)],

with analogous expressions for binary complexes. Importantly, the reported energy term is the CO_2_ interaction (complexation) energy rather than a true desorption or regeneration energy. The latter requires free-energy–based sampling methods to capture entropic effects and kinetic barriers associated with CO_2_ release. Therefore, accurate estimation of regeneration energetics is beyond the present DFT scope and will be addressed in future work using enhanced sampling molecular dynamics and higher-level quantum-chemical approaches.

Detailed wavefunction analyses were performed using the Multiwfn (version 3.7) program [29] to compute reduced density gradient (RDG) functions, non-covalent interaction (NCI) plots, critical point (CP) features (based on electron density topology), and an electron localization function (ELF) map. Visual representations of RDG, and CP isosurfaces and interaction regions were generated using VMD (version 1.9.1) software [30], enabling clear identification of hydrogen bonding, electrostatic interactions, and dispersion-dominated regions involved in CO_2_ fixation.

## 4. Conclusions

In this study, we performed a comprehensive DFT investigation using the B3LYP/6-311+G(d,p) level of theory combined with wavefunction (MEP, RDG/NCI, QTAIM, and ELF) and interaction energy analyses to elucidate the molecular-level mechanisms of CO_2_ absorption by a hydroxy-containing amidinium chloride ionic liquid supported by a polystyrene oligomer.

Our findings demonstrate that the PS support does not act as an inert matrix but actively enhances CO_2_ binding through cooperative non-covalent interactions. MEP maps revealed that PS induces favorable polarization of the IL anion, the primary CO_2_ binding site. RDG/NCI and QTAIM analyses confirmed the presence of Cl^−^⋯π and C-H⋯π interactions between PS and the IL, while ELF analysis showed enhanced electron localization in the Cl-CO_2_ bond within the ternary complex compared to the isolated IL. The ternary PS–IL–CO_2_ complex shows a substantially enhanced interaction energy compared to the isolated IL–CO_2_ complex, quantitatively confirming the cooperative role of the PS support. Collectively, these results demonstrate that PS-supported amidinium ILs show enhanced CO_2_ binding through synergistic electrostatic and dispersion contributions, providing atomic-scale insights for the design of hybrid polymer–IL absorbents.

Future work should focus on extending these computational analyses to longer-chain PS polymers and alternative IL anions (e.g., tetrafluoroborate, hexafluorophosphate, or acetate) to optimize CO_2_ capacity and selectivity. Experimental validation via synthesis, gas adsorption isotherms, and spectroscopic characterization (FTIR and NMR) of the predicted ternary complexes is urgently needed. Additionally, molecular dynamics simulations could assess the dynamic stability and diffusion behavior of CO_2_ within the polymer–IL matrix under realistic operating conditions, while machine learning approaches may accelerate the discovery of optimal polymer–IL combinations.

## Figures and Tables

**Table 1:** Overview of recent studies on hydroxy containing amidine for carbon dioxide capture.

Study Focus	Methodology	Key Findings
Methanol-mediated CO_2_ fixation by hydroxy amidine to form poly IL [10].	FTIR, ^1^H/^13^C NMR, viscosity/mass measurements, DFT calculations, XPS/SEM/TGA.	Methanol enables H-bonding activation for amidinium/alkyl-carbonate formation and polymerization; DFT shows reduced HOMO–LUMO gap.
Reversible CO_2_ capture/release by linear amidine copolymers [11].	Click chemistry for amidine grafting, TGA for adsorption cycles, neutron vibrational spectroscopy, DFT.	22 wt% CO_2_ capacity at 27 °C, release at 50 °C; strong CO_2_ bending on amidine; hydrolysis reduces capacity to 1.7 wt%.
CO_2_ fixation by bicyclic amidines (DBU, DBN) forming zwitterions [12].	Solution reactions, ^13^C NMR, theoretical calculations on orbital control.	Zwitterionic N-CO_2_ complexes; DBU shows enzyme-like CO_2_ transfer to amines; thermal stability correlates inversely with activity.
CO_2_ capture by cyclic guanidine/amidines like DBU/TBD [13].	Bubbling CO_2_ in CH_3_CN, NMR/TGA/elemental analysis.	Zwitterion vs. bicarbonate formation; efficient capture/release at ambient conditions.
Dry CO_2_ reaction with hydroxyalkyl amidines on supports [14].	Quantitative absorption/release near RT.	Near-quantitative fixation under dry conditions; moderate heating for release.
Mechanisms of CO_2_ with amines/amidines/water [15].	Six-membered ring pathway modeling.	Nucleophilic roles lead to carbamates/bicarbonates; unified for all interactions.
Atomistic study of PS-supported amidinium chloride IL for CO_2_ absorption (this work).	DFT (B3LYP/6-311+G(d,p)), MEP, RDG/NCI, QTAIM, ELF, and interaction energy.	PS support actively enhances CO_2_ binding. PS polarizes the IL anion, increasing its nucleophilicity and stabilizing the bent CO_2_ geometry through synergistic electrostatic and dispersion forces.

**Table 2:** Optimized structural parameters for IL–CO_2_, PS–CO_2_, and PS–IL–CO_2_ complexes. Bond lengths (Å) are averaged values from extracted optimized coordinates.

System	Cl⋯C (Å)	Cl⋯H-N (Å)	N-H⋯O (Å)	C-H⋯O (Å)	C-O (Å)	Comparison with Previous Work
IL–CO_2_	3.22	2.47	2.22	2.85	1.21	In previous work, it was found that IL alone captures CO_2_ and forms alkyl-carbonate zwitterionic adducts stabilized by H-bonds [9,16,17].
PS–CO_2_	-	-	-	3.56	1.17	
PS–IL–CO_2_	3.18	2.51	2.24	2.92	1.23	

**Table 3:** Interaction energies (*ΔE*, kcal/mol) for binary and ternary complexes calculated at the B3LYP/6-311+G(d,p) level of theory.

Complex	*E* (CO_2_)	*E* (IL)	*E* (PS)	*E* (Complex)	*ΔE*
IL–CO_2_	−495,292.47	−2,506,382.46	-	−3,001,688.65	−13.72
PS–CO_2_	−495,292.47	-	−3,256,197.35	−3,751,491.58	−1.75
PS–IL–CO_2_	−495,292.47	−2,506,382.46	−3,256,197.35	−6,257,907.28	−35.00

## Data Availability

All data supporting the findings of this computational study are reported in the manuscript.

## Supplementary Materials

The following supporting information can be downloaded at: https://www.mdpi.com/article/10.3390/molecules31132360/s1, optimized structure (Figure S1), MEP map (Figure S2), RDG/NCI plot (Figure S3), and QTAIM critical points (Figure S4) of the ternary PS/IL/CO_2_ complex, provided as high-resolution versions for improved readability.

## Abbreviations

The following abbreviations are used in this manuscript:

CO_2_	Carbon dioxide
CP	Critical point
DFT	Density functional theory
ELF	Electron localization function
ILs	Ionic liquids
MEPs	Molecular electrostatic potential maps
NCIs	Non-covalent interactions
PS	Polystyrene
RDG	Reduced density gradient
