# Supplementary material for: Atomistic Study of Polystyrene Supported by Amidinium-Based Ionic Liquid for CO2 Absorption
Source: Molecules. 2026 Jul 4;31(13):2360. doi: 10.3390/molecules31132360 (PMC13363479; doi:10.3390/molecules31132360)
Supplement: Supplementary file 1 [file molecules-31-02360-s001.zip › molecules-4393064-supplementary.pdf]

Supporting material

Atomistic Study of Polystyrene Supported by Amidinium-Based Ionic Liquid for CO_2 Absorption

Irina Irgibaeva [1,*], Anuar Aldongarov [1,*], Lyazzat Abulyaissova [2], Abzal Taltenov [1], Damen Nurgaliyeva [3], Mirat Karibayev [4], Saparbek Tugelbay [4], Farkhad Tarikhov [4], Yerbolat Tashenov [1] and Nikolay Barashkov [5]

1 Department of Chemistry, L.N. Gumilyov Eurasian National University, Astana 010000, Kazakhstan; abzal06@mail.ru (A.T.); tashenovyerbolat@gmail.com (Y.T.)
2 Department of Chemistry, Buketov National Research University, Karaganda 100028, Kazakhstan; abu.lyazzat@gmail.com
3 Department of Natural Sciences, Astana International University, Astana 010000, Kazakhstan; damennurgalieva@gmail.com
4 National Laboratory Astana, Nazarbayev University, Astana 010000, Kazakhstan; mirat.karibayev@nu.edu.kz (M.K.); saparbek.tugelbay@nu.edu.kz (S.T.); farkhad.tarikhov@nu.edu.kz (F.T.)
5 Micro-Tracers, Inc., 1370 Van Dyke Avenue, San Francisco, CA 94124, USA; nikolay@microtracers.com
* Correspondence: irgsm@mail.ru (I.I.); enu-2010@yandex.kz (A.A.)

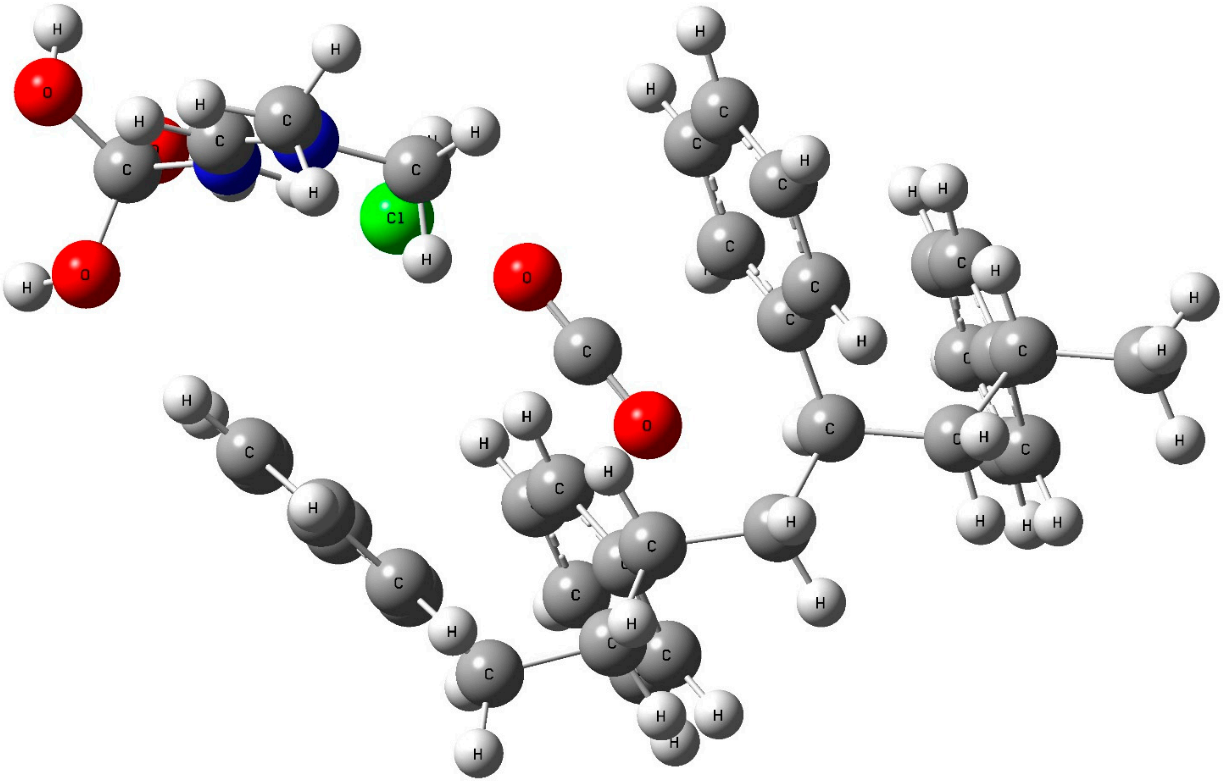

Figure S1: Optimized structure of PS oligomer supported by hydroxy-containing [Amim]Cl IL in the presence of CO_2. Color scheme: dark grey (carbon); white (hydrogen); red (oxygen); blue (nitrogen); green (chloride).

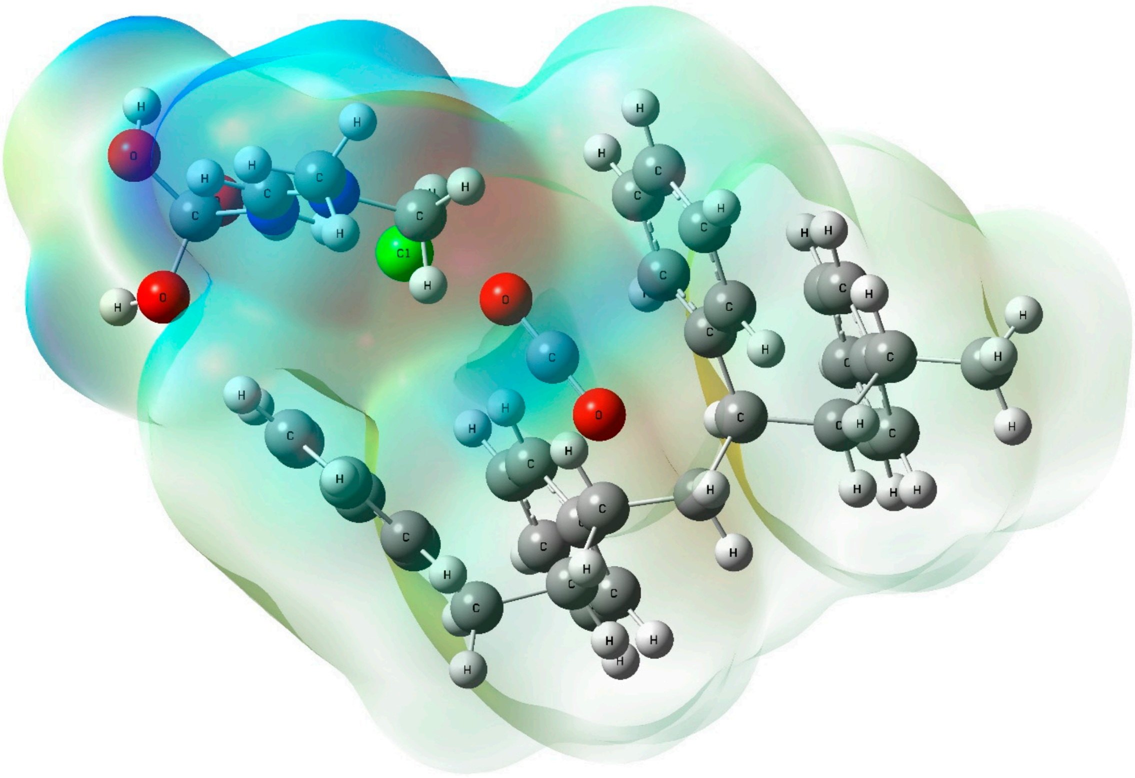

Figure S2. MEPs of PS oligomer supported by hydroxy-containing [Amim]Cl IL in the presence of CO_2. Color scheme: dark grey (carbon); white (hydrogen); red (oxygen); blue (nitrogen); green (chloride).

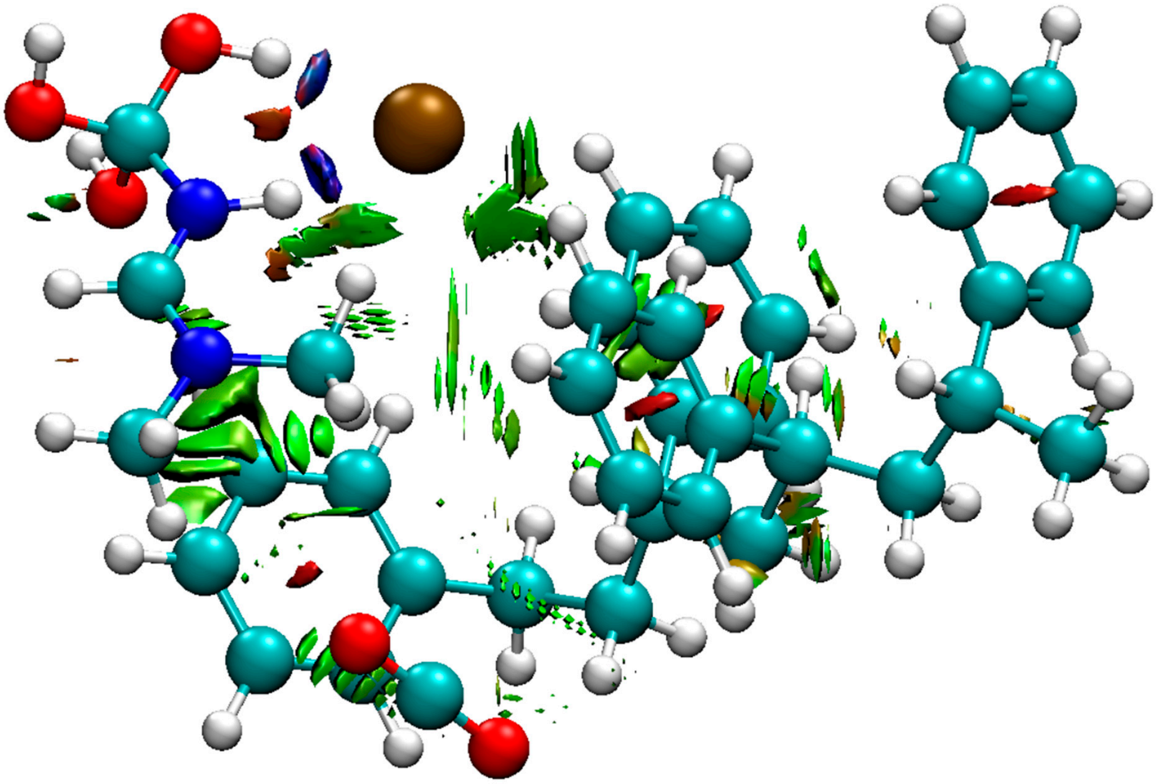

Figure S3. RDGs and NCIs of PS oligomer supported by hydroxy-containing [Amim]Cl IL in the presence of CO_2. Color scheme: cyan (carbon); white (hydrogen); red (oxygen); blue (nitrogen); dark brown (chloride).

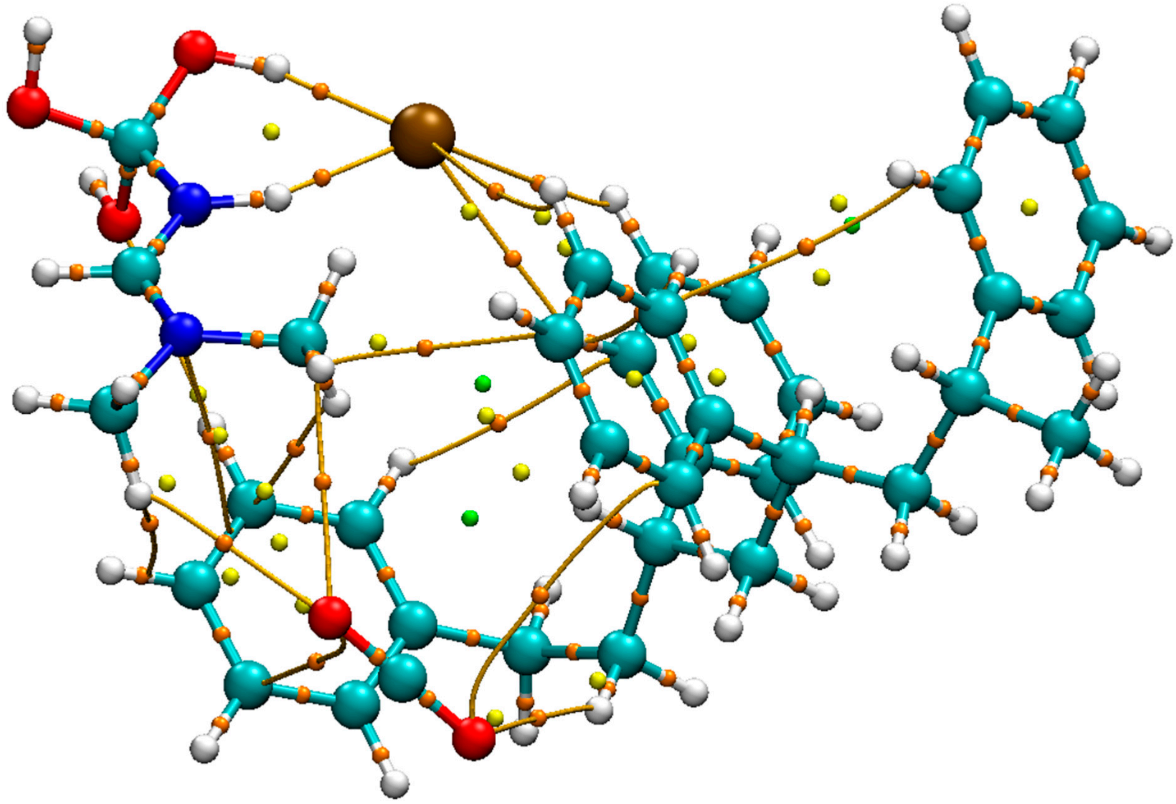

Figure S4. Critical points of PS oligomer supported by hydroxy-containing [Amim]Cl IL in the presence of CO_2. Color scheme: cyan (carbon); white (hydrogen); red (oxygen); blue (nitrogen); dark brown (chloride). The yellow paths and small yellow dots in the figure indicate critical points.
